# UCH-L1 and GFAP Serum Levels in Neonates with Hypoxic–Ischemic Encephalopathy: A Single Center Pilot Study

**DOI:** 10.3389/fneur.2014.00273

**Published:** 2014-12-19

**Authors:** Martha V. Douglas-Escobar, Shelley C. Heaton, Jeffrey Bennett, Linda J. Young, Olena Glushakova, Xiaohui Xu, Daphna Yasova Barbeau, Candice Rossignol, Cindy Miller, Alissa M. Old Crow, Ronald L. Hayes, Michael D. Weiss

**Affiliations:** ^1^Department of Pediatrics, University of Florida, Gainesville, FL, USA; ^2^Department of Pediatrics, University of California San Francisco, San Francisco, CA, USA; ^3^Department of Clinical and Health Psychology, University of Florida, Gainesville, FL, USA; ^4^Department of Radiology, University of Florida, Gainesville, FL, USA; ^5^Department of Statistics, University of Florida, Gainesville, FL, USA; ^6^Banyan Biomarkers, Inc., Alachua, FL, USA; ^7^Department of Biostatistics, University of Florida, Gainesville, FL, USA

**Keywords:** biomarkers, HIE, UCH-L1, GFAP

## Abstract

**Objective:** We examined two potential biomarkers of brain damage in hypoxic–ischemic encephalopathy (HIE) neonates: glial fibrillary acidic protein (GFAP; a marker of gliosis) and ubiquitin C-terminal hydrolase L1 (UCH-L1; a marker of neuronal injury). We hypothesized that the biomarkers would be measurable in cord blood of healthy neonates and could serve as a normative reference for brain injury in HIE infants. We further hypothesized that higher levels would be detected in serum samples of HIE neonates and would correlate with brain damage on magnetic resonance imaging (MRI) and later developmental outcomes.?

**Study Design:** Serum UCH-L1 and GFAP concentrations from HIE neonates (*n* = 16) were compared to controls (*n* = 11). The relationship between biomarker concentrations of HIE neonates and brain damage (MRI) and developmental outcomes (Bayley-III) was examined using Pearson correlation coefficients and a mixed model design.

**Result:** Both biomarkers were detectable in cord blood from control subjects. UCH-L1 concentrations were higher in HIE neonates (*p* < 0.001), and associated with cortical injury (*p* < 0.055) and later motor and cognitive developmental outcomes (*p* < 0.05). The temporal change in GFAP concentrations during (from birth to 96 h of age) predicted motor developmental outcomes (*p* < 0.05) and injury to the basal ganglia and white matter.

**Conclusion:** Ubiquitin C-terminal hydrolase L1 and GFAP should be explored further as promising serum biomarkers of brain damage and later neurodevelopmental outcomes in neonates with HIE.

## Introduction

Hypoxic–ischemic encephalopathy (HIE) is a serious birth complication due to systemic asphyxia (1), which occurs in about 20 of 1,000 full-term infants and nearly 60% of very low-birth weight (premature) newborns (2–4). Between 10 and 60% of babies who exhibit HIE die during the newborn period (4). Of the surviving neonates with HIE, up to 25% have permanent neurodevelopmental handicaps in the form of cerebral palsy (CP), mental retardation, learning disabilities, or epilepsy (5–7). Until recently, treatment of HIE consisted of supportive care including respiratory support, treatment of hypotension, careful monitoring of fluid and electrolytes, and treatment of seizures. In the last decade, research has shown that therapeutic hypothermia improves the neurological and neurodevelopmental outcome of a subgroup of infants with moderate HIE (8–11). Since, more than 47% of treated infants are non-responders to hypothermia (10), we should strive for a better patient stratification including time, location and severity of brain lesion. To be effective, hypothermia should be initiated as soon as possible and no later than 6 h after the initial insult (9, 12). Unfortunately, the bedside clinician is not currently able to accurately identify the neonate who will respond versus the non-responder because accurate clinical indicators cannot be assessed during treatment due to sedatives administered and the effects of hypothermia itself. Therefore, the development of a new, rapid, and reliable prognostic test is essential for making therapeutic decisions.

Current monitoring and evaluation of HIE, outcome prediction, and efficacy of hypothermia treatment rely on a combination of a neurological exam, ultrasound, magnetic resonance imaging (MRI), and electroencephalography (EEG) (13–17). However, these methods do not adequately identify hypothermia non-responders. MRI requires transport of the neonate with a requisite 40–45 min scan, which is not appropriate for unstable neonates. The amplitude integrated EEG (aEEG), is a helpful bedside monitoring technique for seizures and predict HIE outcomes. However, hypothermia depresses the aEEG and thus limits its early predictive ability. Improvement in aEEG tracings may be delayed until the patient is rewarmed and no longer on sedation (18, 19). Consequently, the development of a simple, inexpensive, non-invasive, rapid biochemical test is essential to identify severity of brain injury, distinguish responders from non-responders to hypothermia and assess outcome.

Although many potential biomarkers of brain damage exist, glial fibrillary acidic protein (GFAP) and ubiquitin C-terminal hydrolase L1 (UCH-L1) hold significant promise in this population. *GFAP* is a type III intermediate filament that forms part of the cytoskeleton of mature astrocytes and other glial cells but is not found outside the CNS (20). CNS injury that causes gliosis and subsequently up-regulates GFAP makes GFAP an attractive candidate biomarker for brain injury screening. *UCH-L1*, a highly abundant neuronal protein, is thought to play a critical role in cellular protein degradation during both normal and pathological conditions (21). Both pre-clinical and clinical studies showed that UCH-L1 levels were elevated in CSF and serum following TBI and stroke in a manner significantly associated with measures of injury severity and outcome (22–28). A recent pilot study in neonates with HIE found that serum GFAP but not UCH-L1 correlated with motor outcomes (29).

In this study, we examined the levels of GFAP and UCH-L1 in cord blood to establish normative levels in cord blood. We next examined the level of UCHL-1 and GFAP in patients with HIE undergoing hypothermia at several time points. We hypothesized that the serum concentrations of these two select proteins would be (1) detectable in cord blood in neonates, (2) the levels of UCH-L1 and GFAP would be elevated in the patients with HIE compared with controls, (3) higher in neonates with HIE undergoing hypothermia who had worse brain MRI, and (4) higher HIE patients with a worse developmental outcomes (Bayley-III). Comparisons were then made between the volume of injury as measured by MRI and neurodevelopmental outcomes as measured by formal developmental testing.

## Materials and Methods

### Patient populations

All aspects of this study were approved by the University of Florida Institutional Review Board, and patients were enrolled after obtaining the informed consent from the parents, at Shands Teaching hospital at the University of Florida Health (2012–2013). All studies were approved by the Institutional Review Board at the University of Florida.

#### Control population

Cord blood samples were obtained from healthy neonates who did not have any prenatally diagnosed known risk factors for HIE. The neonates had Apgar scores of 8 or higher at 1 min and 8 or higher at 5 min. In addition, none of the controls had abnormal physical examination or were admitted to the NICU.

#### HIE population

Patients with HIE who were eligible for hypothermia therapy were recruited. Entry criteria for hypothermia included a gestational age of 35 weeks or greater, birth weight of 1.8 and ≤6 h of age. The neonates had evidence of encephalopathy as defined by seizures or abnormalities on a modified Sarnat exam (level of consciousness, spontaneous activity, posture, tone, primitive reflexes including suck and Moro, autonomic system findings including pupils, heart rate, and respirations). Evidence of hypoxic–ischemic injury as defined by a pH of 7.0 or less and/or a base deficit of <16 or a pH between 7.01 and 7.15 and/or a base deficit between 10 and 15.9 or no blood gas available and an acute perinatal event (cord prolapsed, heart rate decelerations, uterine rupture) (Table 1).

**Table 1:** **The patient demographics of the 16 subjects with HIE are shown**.

Gestational age	38 ± 2 weeks
Transferred	44%
NRFHT	55%
**Apgar scores**
5 min	2 ± 2
10 min	3 ± 2
Intubation in DR	81%
Cord pH	6.98 ± 0.16
Base deficit	−18 ± 6
**Sarnat stage**
Moderate	41%
Severe	58%
Inotropic support	50%
EEG seizures	19%

### Blood sample processing

Blood (1 ml) was collected using a tiger top 3.5 ml serum separator tube (BD Vacutainer SST Plus Blood Collection Tube). Samples were allowed to clot upright at room temperature for 30 min in processing lab (45 ± 15 min from time of collection), then spun at 1200 RCF (g) at room temperature for 15 min if fixed angle centrifuge rotor. Spun serum was then collected and transferred using a disposable transfer pipette into a 2 ml cyrovials with red cap inserts (USA Scientific REF 1420-9705). A fiberboard cryogenic storage box (Fisher Part No. 03-395-114 or equivalent) was used to store spun serum aliquots. The samples were then stored in −80°C freezer. The samples were stored (until all samples were collected) and then hand-carried in dry ice, from our laboratory to Banyan Biomarkers to be process immediately upon arrival.

### Enzyme-linked immunosorbent assay

Blinded serum samples were processed at Banyan Biomarkers, Incusing proprietary sandwich enzyme-linked immunosorbent assays (ELISAs) to determine the concentrations and temporal profiles of UCH-L1 and GFAP in human serum. Banyan has successfully used these sensitive biomarker assays in a series of previously published studies in adults following TBI and epilepsy (24–26, 30–32). A detailed description of the ELISA procedures has been published elsewhere (31). Briefly, both mouse monoclonal capture antibody against recombinant UCH-L1 full length and partial protein, and rabbit polyclonal detection antibody were produced in-house at Banyan Biomarkers, Inc. Similarly, proprietary mouse monoclonal antibody for solid phase immobilization and a polyclonal rabbit detection antibody were used for ELISA to detect the levels of intact GFAP and its breakdown products. Such an approach allows more sensitive detection of GFAP analytes from patients’ blood (31, 33). Standard curves using recombinant proteins were generated for each assay and quantitative determination of the biomarker levels in unknown samples were based on four-parameter non-linear regression analyses using SigmaPlot software (Systat, Chicago, IL, USA).

### MRI scoring and volumetric analysis

Magnetic resonance imaging was performed between 4 and 12 days of age since the majority of the patients are stable enough for transport. All patients were scanned on the same 3 T scanner (Verio; Siemens, Erlangen, Germany), with a 32-channel head coil. Analysis focused on the T1-weighted, T2-weighted, and diffusion weighted (DWI) abnormalities. A single subspecialty board-certified neuroradiologist with 10 years of experience in neonatal imaging interpreted all the MRI images using the Barkovich scoring system (34). Brain injury was stratified according to location into four groups: cortical, basal ganglia and thalamus, deep white matter. The volumetric T1-weighted images (3D MP-RAGE), with effective voxel size of 1 mm × 1 mm × 1 mm were analyzed using ITK-SNAP Version 2.0 (Penn Image Computing and Science Laboratory). While correlating with DWI and standard T1- and T2-weighted images, the area of abnormality was manually traced on each slice. The volume of abnormality was then calculated automatically by the software.

### Statistical analysis

All statistical analyses were performed using SAS 9.3 (Cary, NC, USA). To compare levels of UCH-L1 and GFAP either between HIE and control neonates or HIE neonates over time, a generalized linear model was fit using a logarithmic link function so that the assumption of normality of the residuals was approximately met. This provides a comparison of medians on the data scale. In addition, the differences in variability among neonates over time and the correlation among measurements from the same neonate were accounted for in the modeling process. The Pearson correlation coefficient was used to assess the association between each of the protein biomarkers (UCH-L1 and GFAP) and percent injury in the cortex, white matter, and basal ganglia regions as measured by MRI and the cognitive, language, and motor developmental outcomes. Receiver–operator characteristic (ROC) curves were constructed to determine area under the curve (AUC) for each serum biomarker value obtained from each of the time points sampled to with the ability to detect HIE. The graphs were created using GraphPad Prism (GraphPad Software, La Jolla, CA, USA).

### Developmental testing

Neurodevelopmental outcome of the HIE infants was assessed between 4.8 and 10 months of age using the Bayley Scales of Infant and Toddler Development, Third Edition (35). The three primary Bayley-III Index Scores (cognitive, language, and motor) were used to classify HIE participants into “good outcome” and “poor outcome” groups. All raw scores were transformed into norm-referenced standard scores (scale mean = 100 with SD = 15) using the Bayley-III scoring software published with the test. Standardized scores that were at or >1 SD below the normative sample mean (i.e., scores ≤ 85) were classified as indicative of “poor outcome” (i.e., developmental delay in one or more domains).

## Results

### UCH-L1 and GFAP in umbilical cord blood

**Figure 1:**
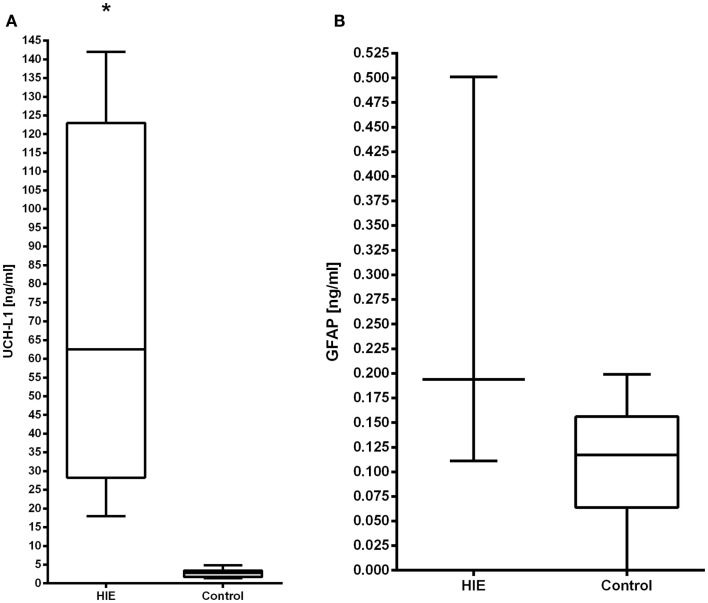
**The serum concentrations of UCH-L1 (A) and GFAP (B) in neonates with HIE are compared with control neonates**. Increased serum levels of UCH-L1 at 0–6 h in HIE patients (*n* = 4) compared with controls (*n* = 11), *p* < 0.0001. The serum concentration of GFAP did not differ statistically from the control population.

A total of 11 patients had cord blood collected. Both UCH-L1 and GFAP were able to be detected in the serum samples from cord blood (Figures 1A,B).

### HIE subjects

The demographics of the HIE patients are shown in Table 2. A total of 16 subjects underwent hypothermia and had serum samples obtained. A total of 54 samples were obtained for analysis.

**Table 2:** **Demographic and key medical variables in a prospective sample of HIE neonates treated with hypothermia who had serial biomarker samples obtained and formal developmental follow-up**.

Patient	Biomarker profile						Neurodevelopmental outcome				
	UCHL (ng/ml)			GFAP (ng/ml)			(Bayley-III assessment)				
	0–6 h	12 h	24 h	0–6 h	12 h	24 h	Age (months)	Cognitive	Language	Motor	Outcome
Subject 1	141.71	–	10	0.2	–	0.06	9.9	130	106	82	Poor
Subject 2	–	–	1.792	0	–	NA	10	135	121	100	Good
Subject 3	17.82	2.281	2.308	0	0.09	0.1	9.7	105	100	94	Good
Subject 4	–	–	1.296	–	–	0.03	5.5	145	91	79	Poor
Subject 5	–	59.43	–	–	0.065	–	6.1	85	103	46	Poor
Subject 6	66.18	36.65	15.96	0.501	0.369	0.218	4.8	85	83	82	Poor

*Bayley-III index scores (scale mean = 100; SD = 15); –, samples were unavailable*.

### Receiver–operator characteristic

**Figure 2:**
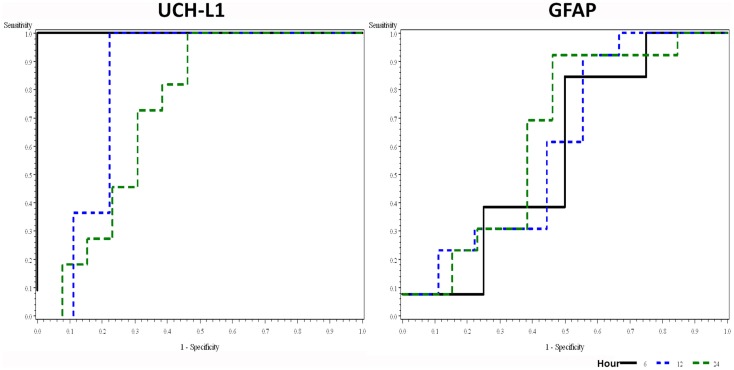
**Receiver–operator characteristic plots of UCH-L1 and GFAP at different time point in detecting HIE**. Area under curve (AUC with 95% confidence interval) for UCH-L1 is 1.00, 0.83 (0.57–1.00), and 0.73 (0.51–0.94) for 0–6, 12, and 24 h, respectively. AUC (95% CI) for GFAP is 0.58 (0.15–1.00), 0.61 (0.33–0.88), and 0.64 (0.41–0.87) for 0–6, 12, and 24 h, respectively.

We analyzed the ability of UCH-L1 and GFAP to detect HIE using values measured at different time points (Figure 2). The ROC plots showed that UCH-L1 measured from 0 to 6 h after the birth had AUC = 1.00, and there is a decreasing trend of AUC with the time of measurement. AUC summarizes diagnostic accuracy, with those approaching 1.00 being very accurate while AUC approaching 0.5 are considered more associated with pure chance. The AUC for GFAP increased slightly over time, with all point estimation >0.5.

### Concentrations of UCH-L1 and GFAP in serum of neonates with HIE compared with cord blood at 0–6 h after birth

The serum levels of UCH-L1 at 0–6 h of age (*n* = 4) were compared with age-matched controls (*n* = 11). The results demonstrate that UCH-L1 levels are significantly higher in the HIE group compared with the controls (*p* < 0.001). Notably, the lowest concentration in the HIE patients was 18 ng/ml compared to the highest value of 4.8 ng/ml in the controls (Figure 1A). The levels of GFAP were not significantly elevated compared to control cord blood samples at 0–6 h of age (*p* = 0.7) (Figure 1B).

### Serum concentrations of UCH-L1 and GFAP over time neonates with HIE

**Figure 3:**
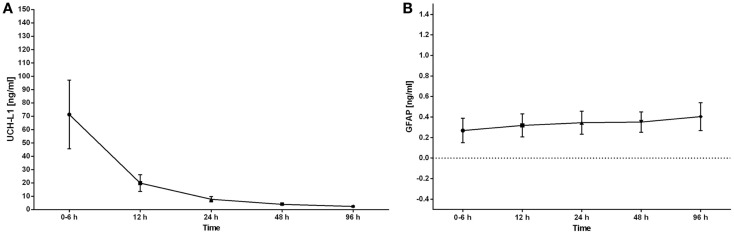
**The serum concentrations of UCH-L1 and GFAP in neonates with HIE are plotted over the time of sample collection**. The serum concentrations are expressed as the mean ± SEM. The serum concentration of UCH-L1 decreased rapidly over the initial 24 h with the highest concentrations obtained at 0–6 h **(A)**. The serum concentrations of GFAP increased over the 96 h of sampling **(B)**.

The serum concentrations of UCH-L1 were still above control concentrations at 12 h (*p* < 0.05). By 24 h, there was no difference between the control concentrations and the HIE concentrations. The concentration of UCH-L1 dropped significantly between the 0–6 h and 12 h sampling time points. The 0–6 h sampling time point was significantly higher than all other sampling time points (Figure 3A). The serum concentrations of GFAP demonstrated a trend over time with a rise in the concentration over the 96 h measured (Figure 3B).

### Correlation of serum UCH-L1 and GFAP concentrations and MRI in neonates with HIE

The serum concentrations of GFAP demonstrated the strongest correlations with the percent injury of the cortex at a time of 0–6 h of age (*p* = 0.08) and the percent injury of white matter and basal ganglia injury at 12 h of age (*p* = 0.06). UCH-L1 concentrations were associated with cortical injury at 12 h (*p* = 0.055).

### Correlation of serum UCH-L1 and GFAP concentrations and developmental outcomes in neonates with HIE

Developmental outcomes were performed on 6 subjects ranging in age from 4.8 to 10 months of age with an average age of 8 ± 3 months (Table 2). Four of the six subjects had poor developmental outcomes defined as performance on any of the primary Bayley-III domains (motor, cognitive, and language) that was at least 1 SD lower than age-matched normative data. All four subjects who were classified as having poor developmental outcomes exhibited delays in motor development. Two of the four subjects had additional delays in cognitive development, and one subject exhibited delays in all three developmental domains (motor, cognitive, and language).

**Figure 4:**
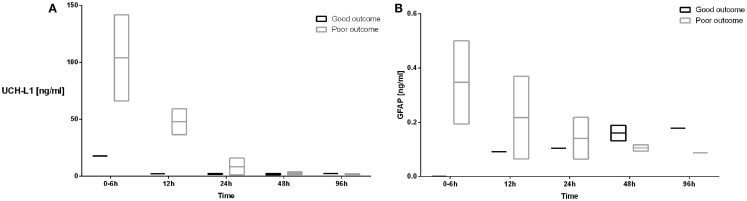
**The serum concentrations of UCH-L1 (A) and GFAP (B) in neonates with HIE who had good (black boxes and lines) and poor developmental outcomes (gray boxes and lines) are plotted over the 96 h sampling period**. The mean serum concentrations are represented by the line while the bar represents the minimum and maximum serum concentrations.

The UCH-L1 profiles in the patient who had a good outcome demonstrated a serum concentration of 17.82 ng/ml at 0–6 h of age with a decrease in the concentration to 2.28 ng/ml at 12 h (Figure 4A). Subjects with a poor developmental outcome had a mean serum concentration of UCH-L1 103 ng/ml at 0–6 h with a decrease to 48 ng/ml at 12 h (Figure 4A). The mean concentration at 24 h was 8.62 for the subjects with a poor developmental outcome and 2.05 in the subjects with a good prognosis.

The concentration of GFAP in the patient who had a good outcome was undetectable at 0–6 h with an increase to 0.092 ng/ml at 12 h. Subjects with a poor outcome had a mean serum concentration of GFAP at 0–6 h of 0.348 ng/ml with a decrease to 0.217 ng/ml at 12 h (Figure 4B).

Subject 6, who exhibited developmental delays across all three domains assessed, had serum concentrations of UCH-L1, which were persistently elevated over the first 24 h. The subject’s concentrations of GFAP were also elevated and were the highest measured values over the first 96 h (Table 2).

Ubiquitin C-terminal hydrolase L1 concentrations at 12 h were correlated with developmental motor outcomes (*p* < 0.05). Further, the temporal changes in UCH-L1 were predictive of both developmental motor (*p* < 0.05) and cognitive outcomes (*p* < 0.05). Finally, the temporal change in the concentration of GFAP was predictive of developmental motor outcomes (*p* < 0.05).

## Discussion

Hypothermia has become standard of care for neonates with HIE. As the field of neonatal neuroprotection evolves, there must be a method to distinguish neonates who will respond to hypothermia to those whom hypothermia will not benefit. A rapid bedside test offers the greatest promise to objectively stratify these neonates. In this report, we have described two proteins, UCH-L1 and GFAP, which are candidates to potentially utilize for stratification. To the best of our knowledge, this is the first report, which has correlated serum biomarkers temporal changes in biomarker concentrations with developmental outcomes.

As a clinical tool, the earlier the proteins can be identified, the better the potential clinical utility. Both proteins were able to be detected in umbilical cord blood samples. Umbilical cord blood measurements were chosen because it is readily obtainable in healthy term neonates and most closely matched the 0–24 h sampling points in our HIE population. The umbilical cord blood provided a true control since the neonates all went to the newborn nursery and did not have serum samples routinely obtained for clinical indications in the first 6 h post birth. Umbilical cord blood may provide information about the degree of injury at the time of birth and potentially the timing of injury based on the serum concentrations at the time of birth. In the future, our group plans to obtain umbilical cord blood from neonates with HIE. The normative information obtained in this study will be used for comparison.

The serum concentrations of UCH-L1 were significantly elevated in the neonates with HIE compared with the control population. Importantly, for the bedside clinician, the elevation in UCH-L1 concentrations occurred at 0–6 h of age and continued to be higher than control concentrations for the first 24 h of sampling. The concentrations of UCH-L1 at 12 h correlated with developmental motor outcomes in neonates with HIE. We suspect that the concentrations at 0–6 h would also predict motor outcomes, but too few samples were obtained to make any firm conclusions. UCH-L1 is an abundant protein localized exclusively to the perikarya and dendrites of neurons (36). Therefore, UCH-L1 may be a very important early marker of neuronal injury. UCH-L1 is resistant to endogenous brain and serum proteases (37). These characteristics along with our results make UCH-L1 an ideal candidate to serve as a biomarker of brain injury in neonates with HIE.

The temporal change in the concentration of UCH-L1 was shown to correlate with developmental motor and cognitive outcomes. Specifically, there was a rapid decrease in the serum concentration in the two neonates with HIE who had good developmental outcomes. This demonstrates that UCH-L1 may be a candidate biomarker to stratify neonate undergoing hypothermia as responders versus non-responders.

The serum concentrations of UCH-L1 at 12 h demonstrated a weak correlation with cortical injury. Our previous work demonstrated that UCH-L1 serum concentrations were higher in neonates with evidence of basal ganglia injury on MRI. It is important to note that these studies were performed in neonates who had not undergone hypothermia therapy. Previously, UCH-L1 and GFAP were shown to have elevated concentrations in neonates with HIE undergoing hypothermia, which correlated with severe MRI abnormalities or death (38).

The blood brain barrier is an anatomic structure composed of brain capillary epithelium joined by tight junctions and the foot processes of astrocytes (39). The blood brain barrier prevents the passive movement of water-soluble molecules larger than 500 Da (39). Following HIE, the blood brain barrier becomes permeable with severe disruption in severe HIE (40). The permeability of the blood brain barrier is measured by CSF to plasma albumin ratios, which may be difficult to perform in an unstable neonate following HIE (40). Serum UCH-L1 has been shown to be a marker of the integrity of the blood brain barrier in patient with traumatic brain injury (39). Therefore, the elevation of biomarker may provide information for the delivery of large neuroprotective drugs, which do not typically cross the blood brain barrier. This may provide a bedside test to determine the exact windows for drug administration, which will individualize care for each patient.

Glial fibrillary acidic protein is a cytoskeleton intermediate filament protein of the astrocytes and is released into the blood following astrocyte death (41). In this report, GFAP concentrations were not higher than controls at 0–6 h but had a higher mean. The concentrations of GFAP in cord blood and at 0–6 h obtained in our study were similar to those previously reported in neonates with HIE undergoing hypothermia (29). Although the concentrations of GFAP were not different than the control samples at 0–6 h, it is important to note that the temporal change in the concentration of GFAP was predictive of developmental motor outcomes.

The serum concentrations of GFAP demonstrated a strong correlation with injury to the cortex (0–6 h), basal ganglia, and white matter (12 h) as detected by MRI. A previous study demonstrated correlations between GFAP concentrations and MRI injury in serum samples obtained at 12 and 24 h of life (38). Taken together, our results along with those published suggest that GFAP may be an important biomarker in predicting regions of brain injury in the first 24 h of life in neonates with HIE.

This study has demonstrated that UCH-L1 is elevated as early as 0–6 h in patients with HIE and the concentrations correlate with developmental motor outcomes. Our data differ from Chalak et al. (42) that found no correlation between UCH-L1 and developmental outcomes, and no temporal changes in UCH-L1 (during hypothermia) (42). Our data demonstrated that the temporal profile of UCH-L1 correlated with the developmental motor and cognitive outcomes. It is possible that our results are different because we had neonates with more severe HIE, making more feasible to demonstrate motor outcomes differences between the groups. Our study’s major weakness was a small number of patients. However, this has been a weakness of all biomarker studies in neonates, to date (43). In addition, the developmental outcomes were performed at a multiple ages post-injury. Although this could be construed as a weakness, this is the only the second study, which has demonstrated that serum based biomarkers correlate with long-term neurologic outcomes and further suggests that serum biomarkers may be used to predict long-term outcomes (42). Furthermore, outcomes were evaluated using norm-referenced scoring methods that control for any inherent differences due to varied outcome time points across subjects. The results from this study provide further data to support the use of UCH-L1 and GFAP in a larger study to evaluate the correlation between serum concentrations and outcomes at 18–24 months of age (42). UCH-L1 appears to offer great promise as a serum based bedside marker to be utilized by the bedside clinician managing neonates with HIE.

## Conflict of Interest Statement

Banyan Biomarkers, Inc., Alachua, FL, USA is commercial company dedicated to the discovery of biomarkers and provided the UCH-L1 and GFAP testing. Banyan Biomarkers received blinded samples and they were not part of the collection or analysis of the result.

## Abbreviations

GFAP, glial fibrillary acidic protein; HIE, hypoxic–ischemic encephalopathy; UCH-L1, ubiquitin carboxyl-terminal esterase L1.
